# A revised taxonomy of Asian snail-eating snakes *Pareas* (Squamata, Pareidae): evidence from morphological comparison and molecular phylogeny

**DOI:** 10.3897/zookeys.939.49309

**Published:** 2020-06-09

**Authors:** Ping Wang, Jing Che, Qin Liu, Ke Li, Jie Qiong Jin, Ke Jiang, Lei Shi, Peng Guo

**Affiliations:** 1 College of Life Science and Food Engineering, Yibin University, Yibin 644007, China Xinjiang Agricultural University Urumqi China; 2 College of Animal Science, Xinjiang Agricultural University, Urumqi 830052, China Yibin University Yibin China; 3 State Key Laboratory of Genetic Resources and Evolution, Kunming Institute of Zoology, Chinese Academy of Sciences, Kunming 650223, China Kunming Institute of Zoology, Chinese Academy of Sciences Kunming China

**Keywords:** Molecular, morphology, new species, snakes, southeast Asia, systematics

## Abstract

The Asian snail-eating snakes *Pareas* is the largest genus of the family Pareidae (formerly Pareatidae), and widely distributed in Southeast Asia. However, potential diversity remains poorly explored due to their highly conserved morphology and incomplete samples. Here, on basis of more extensive sampling, interspecific phylogenetic relationships of the genus *Pareas* were reconstructed using two mitochondrial fragments (cyt b and ND4) and two nuclear genes (c-mos and Rag1), and multivariate morphometrics conducted for external morphological data. Both Bayesian Inference and Maximum Likelihood analyses consistently showed that the genus *Pareas* was comprised of two distinct, monophyletic lineages with moderate to low support values. Based on evidences from molecular phylogeny and morphological data, cryptic diversity of this genus was uncovered and two new species were described. In additional, the validity of *P. macularius* is confirmed.

## Introduction

Pareidae Romer, 1956 is a small family of snakes found largely in Southeast Asia, including the Malay Archipelago, Indo China Peninsula, Bhutan, Bangladesh, India, and China (Zhao 2006; Das 2012; Uetz et al. 2019). It was once considered a subfamily (called Pareatinae) of Colubridae (Smith 1943; Zhao et al. 1998; Zug et al. 2001; Zhao 2006). However, an increasing number of molecular phylogenetic studies have revealed that it is not closely related to the colubrids, and thus has been elevated to family rank (called Pareatidae) (Slowinski and Lawson 2002; Kelly et al. 2003; Lawson et al. 2005; Vidal et al. 2007; Pyron et al. 2013). Recently, Savage (2015) corrected the spelling of Pareatidae to Pareidae. The family Pareidae encompasses 26 species in four genera (*Aplopeltura* Duméril, 1853; *Asthenodipsas* Peters, 1864; *Pareas* Wagler, 1830; and *Xylophis* Beddome, 1878) divided into two subfamilies (Pareinae and Xylophiinae) (Deepak et al. 2018; Uetz et al. 2019).

*Pareas* is the largest genus of Asian snail-eating snakes in Pareidae and contains 14 species (Uetz et al. 2019). Due to its specialized feeding (terrestrial snails and slugs) and foraging behavior, the systematics and evolutionary biology of this group have received much attention in recent years (Hoso and Hori 2008; Hoso et al. 2010; Guo et al. 2011; Vogel 2015; You et al. 2015; Hoso 2017), and considerable progress has been made for resolving *Pareas* systematics (Guo et al. 2011; Pyron et al. 2013; You et al. 2015). For example, based on integrated mitochondrial sequence phylogeny, nuclear haplotype network, and multivariate morphometrics You et al. (2015) explored the taxonomic status of *Pareas* species from Taiwan, including the Ryukyus and adjacent regions. Their results consistently recovered *P. formosensis* Denburgh, 1909 and *P. komaii* Maki,1931 as valid species and *P. compressus* Oshima, 1910 as a junior synonym of *P. formosensis*. In addition, the validity of *P. chinensis* Barbour, 1912 was supported and a new species *P. atayal* You, Poyarkov & Lin, 2015 was described from Taiwan, China (You et al. 2015).

Due to its wide distribution and morphological conservativeness, however, the taxonomy of *Pareas* remains controversial despite the increasing research (Guo et al. 2011; Loredo et al. 2013; Guo and Zhang 2015; Vogel 2015; You et al. 2015). Previous studies on DNA-based phylogeny have indicated that *Pareas* is not monophyletic, but contains two highly supported clades, consistent with scale characters (Guo et al. 2011). However, due to incomplete samples and insufficient morphological data, Guo et al. (2011) deferred making a decision on the division of *Pareas*.

Here, using an integrated taxonomic methods and more extensive sampling, we reconstruct phylogenetic relationships of *Pareas* based on mitochondrial and nuclear DNA, and conducted a morphological comparison between species and populations. Our main goal was to clarify interspecific relationships and explore whether cryptic diversity was present within this diverse Asian snail-eating snakes *Pareas*.

## Materials and methods

### Molecular phylogenetic sampling and sequencing

In total, 52 individuals of *Pareas* representing ten putative species and two unidentified taxa were collected from Southeast Asia through fieldwork or tissue loans from colleagues and museums (Suppl. material 1: Appendix S1). Additional sequences representing 12 species were retrieved from previous studies (Kraus and Brown 1998; You et al. 2015; Figueroa et al. 2016; Deepak et al. 2018). Representatives of *Aplopeltura*, *Asthenodipsas*, and *Xylophis* were also included to investigate the monophyly of *Pareas*.

Total DNA was extracted from liver, muscle or skin preserved in 85% ethanol using an OMEGA DNA Kit (Omega Bio-Tek, Inc., Norcross, GA, USA). The sequences of two mitochondrial gene fragments: cytochrome b (cyt b) and NADH dehydrogenase subunit 4 (ND4), as well as two nuclear genes: oocyte maturation factor mos (c-mos) and recombination activating gene 1 (Rag1) were amplified by polymerase chain reaction (PCR) using primers L14910/H16064 (Burbrink et al. 2000), ND4/Leu (Arèvalo et al. 1994), S77/S78 (Lawson et al. 2005), and R13/R18 (Groth and Barrowclough 1999), respectively. The cycling parameters were identical to those described in the above studies. The double-stranded products were purified and sequenced at Genewiz Co. (Suzhou, China). Sequences were edited and managed manually using SEQMAN in LASERGENE.v7.1 (DNASTAR Inc., Madison, WI, USA), MEGA 7 (Kumar et al. 2016), and GENEIOUS BASIC 4.8.4 (Kearse et al. 2012). For individuals which were detected to be heterozygous in nuclear gene sequences, they were phased using the software program PHASE with default sets of iterations, burn-in, and threshold (Stephens et al. 2001), on the web-server interface SEQPHASE (Flot 2010). One of the phased copies was selected at random to represent each individual in subsequent analyses.

### Phylogenetic analyses

Phylogenetic analyses were conducted using Bayesian inference (BI) and Maximum Likelihood (ML) methods with *Xenodermus javanicus* Reinhardt, 1836, *Gloydius brevicaudus* Stejneger, 1907, and *Lycodon rufozonatus* Cantor, 1842 selected as outgroups based on previous work (Guo et al. 2011; Deepak et al. 2018). Phylogenetic trees were estimated separately for mitochondrial DNA fragments (cyt b and ND4) and nuclear genes (c-mos and Rag1). The best-fit substitution model was selected in PARTITIONFINDER 2.1.1 (Lanfear et al. 2017) with Akaike Information Criterion (AIC).

The BI analyses were performed using MRBAYES 3.2 (Ronquist et al. 2012) with three independent runs of four Markov chains. Each run consisted of ten million generations, started from random trees and sampled every 1 000 generations, with the first 25% discarded as burn-in. Convergence was assessed by examining effective sample sizes and likelihood plots through time in TRACER v1.6 (Rambaut et al. 2014). The resultant trees were combined to calculate Bayesian posterior probabilities (PP) for each node, with nodes of PP ≥ 95% considered strongly supported (Felsenstein 2004). The ML analyses were completed in RAXMLGUI 1.5 (Silvestro and Michalak 2012) under the GTRGAMMA model with 1000 non-parametric bootstraps to replicate topology and assess branch support. Nodes with bootstrap support values (BS) ≥ 70% were considered strongly supported (Hillis and Bull 1993).

Average divergence estimates were calculated from cyt b or ND4 data among congeners under the K2P model with 1 000 bootstraps using MEGA 7 (Kumar et al. 2016).

### Morphological examination

A suite of characters was examined and recorded from 42 voucher specimens (Appendix 1). Except for snout-vent length (SVL) and tail length (TL), which were measured using a measuring tape to the nearest 1 mm, all other characters were measured and recorded following Zhao (2006). For comparison, data for other species were taken from prior published work (Boulenger 1900, 1905; Zhao et al. 1998; Grossmann and Tillack 2003; Guo and Deng 2009; Guo et al. 2011; Loredo et al. 2013; Vogel 2015; You et al. 2015; Hauser 2017).

## Results

### Sequence data

A total of 1 767 (1 095 bp from cyt b, 672 bp from ND4) and 1 635 (612 bp from c-mos and 1 023 bp from Rag1) aligned base pairs were obtained from the two mtDNA fragments and two nuclear genes, respectively. Sequences were translated into amino acids to confirm that no pseudogenes had been amplified. Novel sequences generated were deposited in GenBank (Suppl. material 1: Appendix S1).

### Phylogenetic relationships

The best-fit model selected by PARTITIONFINDER was three-partition (partitioned by codon positions) for both mtDNA and nDNA datasets (Table 1). BI and ML analyses based on two separate datasets depicted consistent topological trees, which are in general accordance with those of Guo et al. (2011) and You et al. (2015).

**Table 1.** The best partition scheme suggested by PARTITIONFINDER 2.1.1 under AIC.

Partition	Model	Partition	Model
cyt b/ND4, position 1	TVM+I+G	c-mos/Rag1, position 1	K81UF+G
cyt b/ND4, position 2	GTR+I+G	c-mos/Rag1, position 2	TVM+I
cyt b/ND4, position 3	TIM+G	c-mos, position 3	TVM+G
		Rag1, position 3	K81UF+G

**Figure 1.**
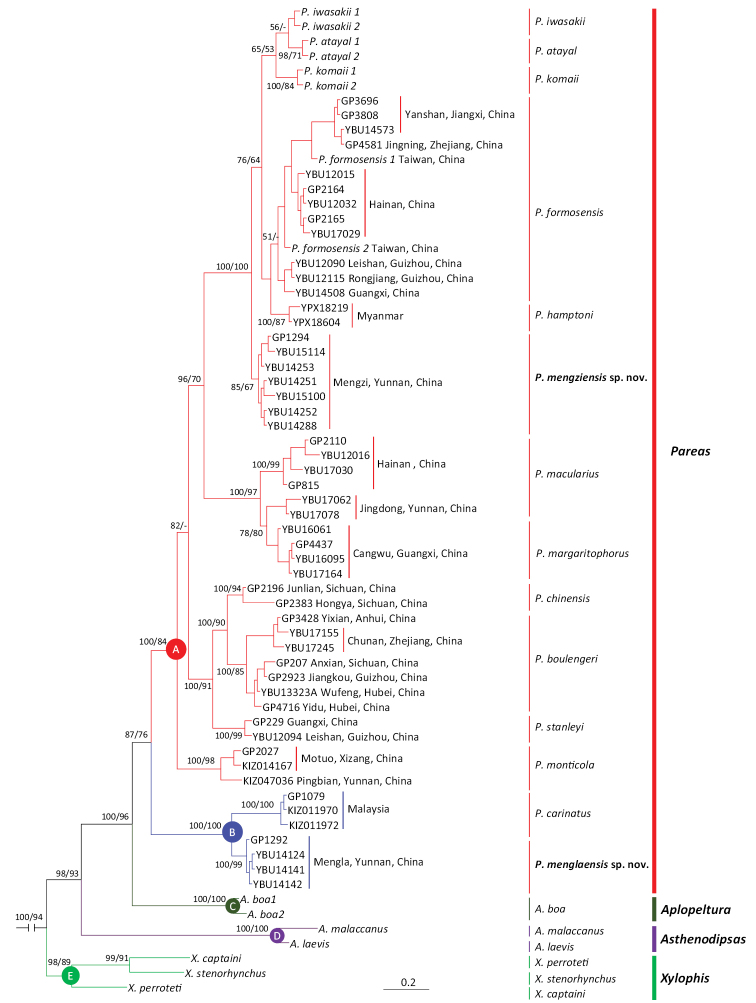
Bayesian inference tree of the Pareidae based on nDNA dataset. Branch support measures are Bayesian posterior probabilities/ML bootstrap support (only where >50%). Branch support indices are not given for most intrageneric nodes to preserve clarity.

**Figure 2.**
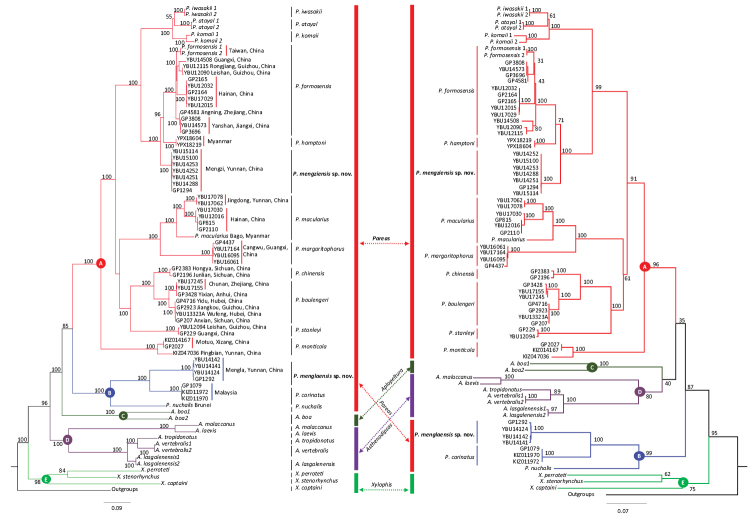
Bayesian inference(left) and Maximum Likelihood(right) trees of the Pareidae based on mtDNA dataset. Branch support measures are Bayesian posterior probabilities and ML bootstrap support respectively. Branch support indices are not given for most intrageneric nodes to preserve clarity.

All analyses strongly supported monophyly of Pareidae as a whole and reciprocal monophyly of *Aplopeltura* (lineage C), *Asthenodipsas* (lineage D), and *Xylophis* (lineage E) (Figs 1, 2).

Monophyly of *Pareas* was supported by either analysis based on mtDNA or nDNA-based BI analysis with moderate support values, and ML analysis based on nDNA data with high support value. Here, *Pareas* consists of two highly supported lineages (A and B). Lineage B is composed of *P. carinatus* Boie, 1828, *P. nuchalis* Boulenger, 1900, and a clade containing four specimens from southern Yunnan, China (Figs 1, 2). Lineage A contains the remaining species, with each putative species and relationships between congeners being highly supported; the specimens from Mengzi, Yunnan, China, formed a well-supported clade, close to *P. hamptoni* Boulenger, 1905.

### Divergence estimates

Table 2 provides the mean K2P divergences among the four lineages (A–D). Lineage A diverged from B by an average genetic distance of 21.3%, which is much higher than that between genera *Aplopeltura* and *Asthenodipsas* (15.1%).

**Table 2.** The average genetic divergence estimates (%, Kimura 2-parameter model with gamma correction) among four lineages (A–D) based on Cyt b.

	lineage A	lineage B	lineage C
lineage A/*Pareas*			
lineage B/*Pareas*	21.3		
lineage C/*Aplopeltura*	18.4	23.0	
lineage D/*Asthenodipsas*	16.9	21.6	15.1

Within lineage A, genetic divergence between species varied from 6.5% (*P. hamptoni* and the population from Mengzi, Yunnan; *P. iwasakii* Maki, 1937 and *P. komaii*) to 29.5% (*P. hamptoni* and *P. margaritophorus* Jan, 1866) based on cyt b and from 8.5% (*P. formosensis* and the population from Mengzi, Yunnan, *P. formosensis* and *P. hamptoni*) to 30% (*P. monticola* Cantor, 1839 and *P. komaii*) based on ND4 (Table 3). Furthermore, the population from Mengzi, Yunnan showed genetic divergences of between 6.5% to 28.8% from the other species.

Within lineage B, the sublineage containing the four individuals from southern Yunnan demonstrated genetic divergences of 18.5% and 26.5% from *P. nuchalis* and *P. carinatus*, respectively, based on the ND4 sequences (Table 3).

**Table 3.** The average divergence estimates (%, Kimura 2-parameter model with gamma correction) of *Pareas* based on cyt b/ND4.

	Taxa	1	2	3	4	5	6	7	8	9	10	11	12	13	14
1	*P. mengziensis* sp. nov.														
2	*P. hamptoni*	6.5/10.7													
3	*P. formosensis*	7.5/8.5	7.1/8.5												
4	*P. komaii*	16.8/22.4	17/25.2	14.6/20.4											
5	*P. iwasakii*	17.4/–	17.2/–	16/–	6.5/–										
6	*P. atayal*	18.5/21.8	18.1/22.3	17.8/20.8	7.8/8.7	8/–									
7	*P. macularius*	23.5/26.4	24.4/26	21.9/27	19.1/28.5	24.4/–	23.4/26.6								
8	*P. margaritophorus*	28.8/25.5	29.5/26.6	26.7/26	23.8/29.5	26.1/–	26.2/29.4	15.5/18.3							
9	*P. boulengeri*	23.3/23.2	23.2/24	19.9/24.6	22.7/26.8	23.3/–	24.9/25.2	21.6/22.5	25.4/23.7						
10	*P. chinensis*	23.4/23.6	24.7/22.2	20.7/22.9	21.5/27.3	24.3/–	25.1/29.1	20.9/24.7	24.6/26.6	8.6/10.6					
11	*P. stanleyi*	28.1/27.7	26.1/30.8	25.4/27.2	21.3/26.4	26.1/–	27/28.3	23.8/22.8	26.9/29	20.9/18.1	19.3/22.3				
12	*P. monticola*	24.4/25.6	24.9/23.1	22.8/24.1	19.6/30	22.7/–	21.7/26.4	19.3/24.5	26.3/26.2	23.7/22.2	23.5/22.9	24.7/28.3			
13	*P. carinatus*	35.8/32.5	37/31.3	36.5/28	34.5/37.9	38.4/–	34.8/37.1	32.4/31.5	36.9/35.2	34.2/33.3	34.5/33.9	39.2/36.7	33.7/29		
14	*P. menglaensis* sp. nov.	35.8/32.3	35.7/30.2	36.1/29.3	35.2/32	38.2/–	35.2/30.7	33.9/31.6	38.8/31.5	35/29.3	39.5/33.9	41.2/33.7	32.4/27.8	18.5/26.5	
15	*P. nuchalis*	–/33.2	–/31.5	–/27.7	–/33.8	–/–	–/32.7	–/32.2	–/34.2	–/34	–/34.8	–/37.9	–/28.7	–/24.8	–/18.5

### Morphological examination

**Figure 3.**
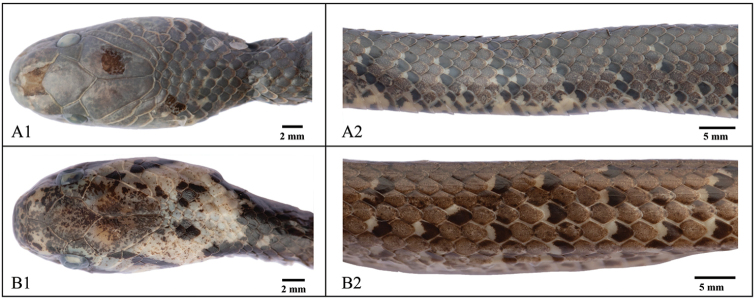
The comparisons of dorsal head (row 1) and median dorsal (row 2) between *Pareas macularius* and *P. margaritophorus*. **A***P. margaritophorus***B***P. macularius*.

A total of 30 characters were measured and recorded for 42 specimens representing seven species and two unidentified taxa of *Pareas* (Appendix 1). Some species or specimens showed markedly different external morphology from their congeners or close relatives. For example, *P. macularius* Theobald, 1868 could be distinguished from *P. margaritophorus* by its keeled dorsal scales (vs. smoothed dorsal scales) (Fig. 3). A detail comparison of morphological characters is listed in Suppl. material 2: Appendix S2 and shown in Suppl. material 4: Figure 1.

The four specimens collected from Mengla County, Yunnan Province, China, were close to those of *P. carinatus*, but could be distinguished from the latter by having 11 rows of strongly keeled dorsal scales at mid-body (vs. 3–5 rows feebly keeled) (Rooij 1917; Smith 1943). The specimens collected from Mengzi, Yunnan Province, China, possessed exclusive characters differed from their congeners, including solid black marking on top of head and dorsal body, three rows of enlarged mi-dorsal scales, and eight or nine infralabials (Suppl. material 4: Fig. 1).

### Descriptions of two new taxa

Multiple studies on species identification and evolution have relied solely on external morphology, which is misguided in reptiles (Guo et al. 2012, 2013, 2014; Xie et al. 2018). In particular, widely distributed species are often proven to be complexes of multiple species (Ukuwela et al. 2013; You et al. 2015; Krysko et al. 2016; Chen et al. 2017; Wang et al. 2019). The snakes of *Pareas* have wide distribution in Asia, its highly morphological conservation has contributed to its frequent misidentification and confusion (You et al. 2015). Morphological comparisons indicated that the specimens collected from Mengzi and Mengla, Yunnan, China were significantly different from their congeners respectively. In addition, the specimens from the two populations were also highly divergent from their closest relatives. Thus, we regarded these specimens as two undescribed taxa.

#### Pareas menglaensis sp. nov.

Taxon classificationAnimaliaSquamata Pareatidae

A947E017-FBEE-5258-8057-64A7A8A36D34

http://zoobank.org/9AB5DAEE-19AA-4A63-8922-713BF1FBFD09

Figure 4

##### Holotype.

YBU 14124, adult female, collected from Mengla County, Yunnan Province, China, at an elevation of 700 m above sea level in June 2014.

##### Paratypes.

YBU 14141 and YBU 14142, two adult males from the same locality as the holotype but collected in July 2012.

##### Diagnosis.

(1) prefrontal separating from orbit; (2) three chin-shield pairs, anterior pair smaller than other two; (3) 9–13 rows of mid dorsal scales keeled; (4) three rows of mid dorsal scales enlarged; (5) single loreal, not bordering orbit; (6) two preoculars, 2–3 suboculars, single postocular; (7) 9–11 temporals (3+3+3, 3+4+4, or 3+4+3); (8) seven supralabials, not bordering orbit; (9) 7–8 infralabials; (10) 3–5 maxillary teeth; (11) cloaca undivided; (12) dorsal scales in 15 rows throughout; (13) 176–177 ventral scales; (14) 65–79 subcaudals, paired.

**Figure 4.**
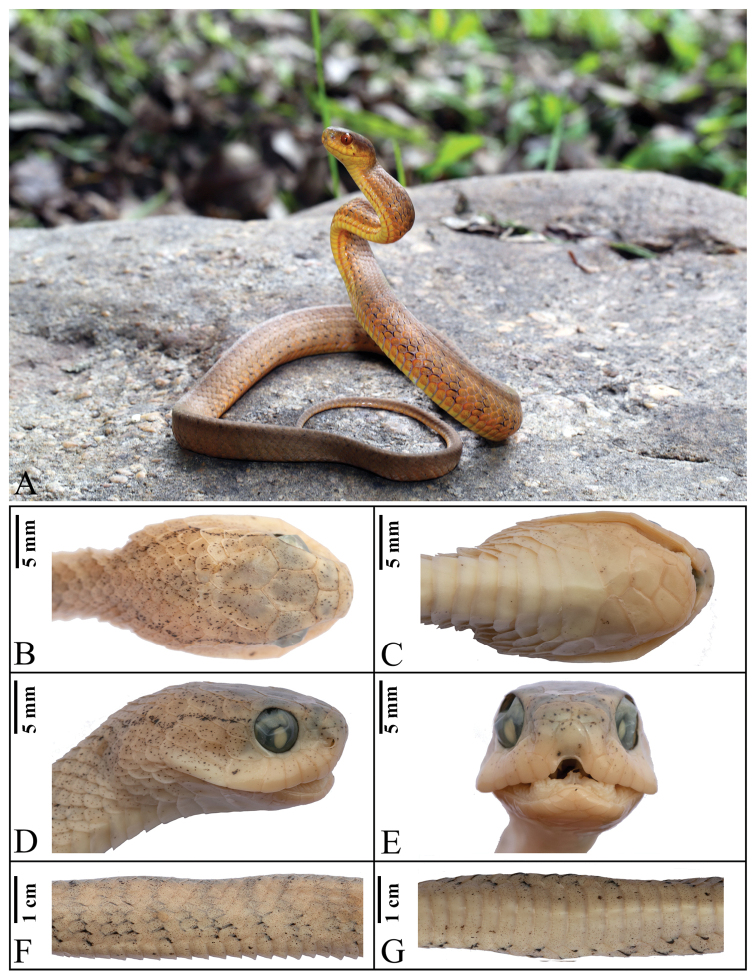
Holotype of *Pareas menglaensis* sp. nov. (YBU 14124). General view(**A**); dorsal (**B**), ventral (**C**), lateral **(D**) and frontal (**E**) views of the head; dorsal (**F**) and ventral (**G**) views of the median body.

##### Description of holotype.

Male, SVL 472 mm, TL 111 mm, TL/total length 0.24; body elongated; snout distinctly blunt; head distinct from neck. Rostral invisible from above, much deeper than broad; nasals undivided. Internasals subtriangular, wider than long; prefrontals pentagonal, length equal to width, not touching eyes; frontal hexagonal, longer than wide; parietals irregular, longer than wide; one supraocular, longer than diameter of orbit; single loreal, separating from eyes; two preoculars; one postocular; two suboculars; nine or ten temporals, 3+4+3 on left and 3+3+3 on right; seven supralabials, not bordering orbit; seven or eight infralabials, first four in contact with anterior chin-shields; three chin-shield pairs, posterior pair larger than other two; ventral scales 177; cloaca undivided; subcaudals 65, paired; dorsal scales in 15 rows throughout, three median rows enlarged, all keeled except for outer two; five maxillary teeth on both sides.

Dorsal surface nearly uniformly light brown with slightly visible black cross-bands. Head light brown with black dusted spots. Thin postorbital stripe extending from postocular to neck. Belly yellowish white, anterior portion without spots except for lateral edges mottled with almost striped dark brown spots, striped spots gradually becoming invisible backwards. Spots and specks on posterior portion of belly appear and become denser later.

##### Description of paratypes.

The paratypes agree in most respects with the description of the holotype. A comparison of the most important morphological characters is summarized in Suppl. material 3: Appendix S3.

##### Etymology.

The specific species is named after the type locality, Mengla County, Yunnan, China. We suggest the common name “Mengla Snail-eating Snake” in English and “Mengla Dun-tou-she” (勐腊钝头蛇) in Chinese.

##### Distribution.

This species is currently known only from the type locality Mengla County, Yunnan, China, with low mountain evergreen broad-leaved forest and a tropical monsoon climate type. It is expected to be found in the surrounding low mountainous areas and in neighboring Laos and Myanmar.

##### Comparison.

*Pareas menglaensis* sp. nov. can be distinguished from *P. carinatus* by 11 rows of dorsal scales strongly keeled at mid-body (vs. 3–5 rows feebly keeled), from *P. nuchalis* by prefrontal separated from orbit (vs. prefrontal bordering orbit), and from all other species of *Pareas* by two or three distinct narrow suboculars (vs. one thin elongated subocular).

#### Pareas mengziensis sp. nov.

Taxon classificationAnimaliaSquamata Pareatidae

9F83DF2E-290B-578E-8887-FBFB69E7B58E

http://zoobank.org/EC677F21-D01B-4C53-998F-D77C7457081B

Figure 5

##### Holotype.

YBU 14252, adult female, collected from Mengzi, Yunnan Province, China, at an elevation of 1 900 m above sea level in July 2014.

##### Paratypes.

Two adult females (YBU 141251 and YBU 15100) and three adult males (YBU 14253, YBU 14288, and YBU 15114) from the same locality and adjacent regions collected in July 2014 and July 2015.

##### Diagnosis.

(1) solid black marking on back of head extending along whole dorsal of body; (2) single preocular; (3) postocular fused with subocular; (4) loreal not bordering orbit; (5) temporals 2+3+3; (6) prefrontal bordering orbit; (7) three rows of mid dorsal scales slightly enlarged; (8) 3–7 rows of mid dorsal scales keeled; (9) 6–7 supralabials; (10) 8–9 infralabials; (11) 6–7 maxillary teeth; (12) cloaca undivided; (13) ventral scales 167–173; (14) subcaudals 54–61, paired.

**Figure 5.**
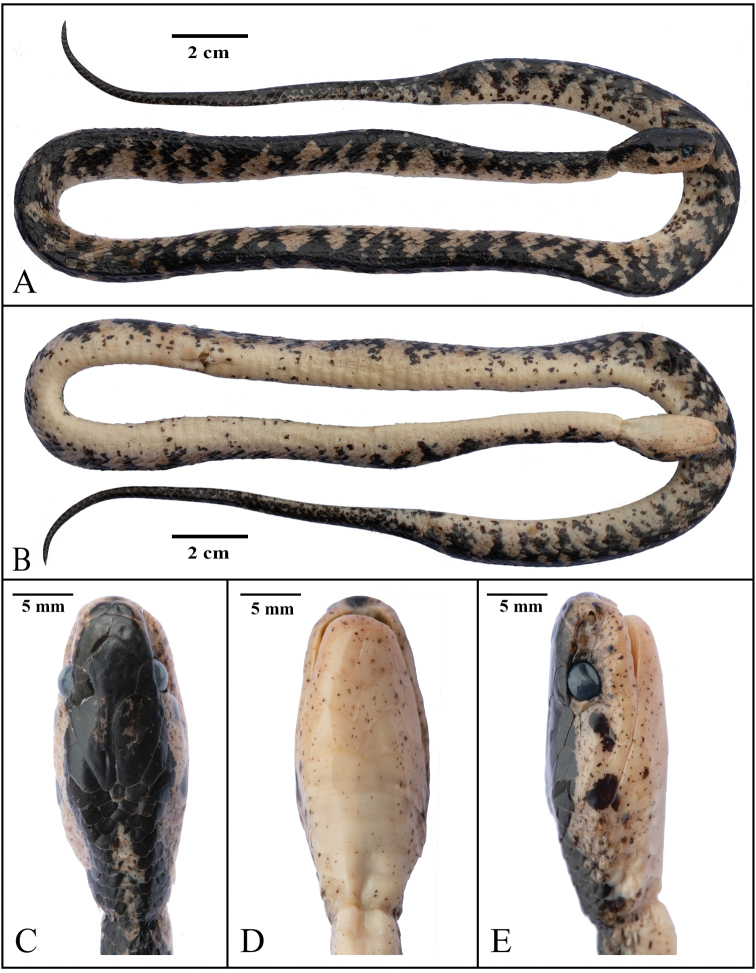
Holotype of *Pareas mengziensis* sp. nov. (YBU 14252). Dorsal (**A**) and ventral (**B**) of general views; dorsal (**C**), ventral (**D**), and lateral (**E**) views of the head.

##### Description of holotype.

Female, SVL 426 mm, TL 98 mm, TL/total length 0.187; body elongated; head distinct from neck. Internasals sub-triangular, wider than long; prefrontals sub-rectangular, wider than long, bordering orbits; frontal shield-shaped; one relatively small supraocular; parietals irregular, longer than wide; rostral almost invisible from above, wider than deep; nasals undivided; single loreal, separating from eyes; single preocular; single thin elongated subocular; postocular fused with subocular, supraocular sub-triangular; temporals 2+3+3; seven supralabials, separating from eyes; 8–9 infralabials, anterior-most in contact with opposite between mental and anterior chin-shields, first four in contact with anterior chin-shields; three chin-shields pairs, anterior pairs larger than other two; ventral scales 170; cloaca undivided; subcaudals 54, paired; dorsal scales in 15 rows throughout, three median rows enlarged, 3–7 rows of mid dorsal scales keeled; 6–7 maxillary teeth.

Solid black marking on back of head extending along whole dorsal of body and tail; sides of head light brownish yellow, speckled with small, irregular, dark brown spots; two black spots on each side of head, anterior one on intersection of anterior two temporals and 6^th^ and 7^th^ supralabials, posterior one on middle of 7^th^ supralabial; vertical brownish yellow stripe on neck, eight scales long and 1–2 scales wide; body brownish yellow with numerous irregular black cross-bands on lateral of body, contacting with solid black dorsal of body, some extending to edges of ventral scales; belly light brown with sparse dark brown spots; tail purely black except for first 20 pairs of subcaudals light brown.

##### Description of paratypes.

The paratypes agree in most respects with the description of the holotype. A comparison of the most important morphological characters is summarized in Suppl. material 3: Appendix S3.

##### Etymology.

The new species is named after the type locality Mengzi City, Yunnan Province, China. We suggest the common name “Mengzi Snail-eating Snake” in English and “Mengzi Dun-tou-she (蒙自钝头蛇)” in Chinese.

##### Distribution.

This species is currently known only from the type locality Mengzi City, Yunnan, China, in deciduous broad-leaved forest with a subtropical monsoon climate. It is expected to be located in the surrounding plateau regions.

##### Comparison.

*Pareas mengziensis* sp. nov. can be distinguished from *P. carinatus*, *P. nuchalis*, and *P. menglaensis* sp. nov. by having one thin elongated subocular (vs. two or three suboculars). It is most similar to *P. yunnanensis* Mell, 1931, *P. niger* Pope, 1928, and *P. nigriceps* in terms of color pattern, but differs from these species by eight or nine infralabials (vs. seven) and three rows of mid dorsal scales enlarged (vs. not enlarged or only one enlarged mid dorsal scale). It differs from the remaining species of *Pareas* by having a large solid black area on back of head and body.

### Validity of *Pareas macularius* Theobald, 1868

Zhao et al. (1998) suggested *Pareas* to be composed of two types of color pattern: color pattern I (*P. macularius* and *P. margaritophorus*) and color pattern II (other species of *Pareas*). *Pareas macularius* was named based on specimens from Martaban, Myanmar. It is distinguished from *P. margaritophorus* by its slightly keeled dorsal scales. However, Huang (2004) held that dorsal scales, keeled or not, are undiagnosable, and thus synonymized *P. macularius* with *P. margaritophorus*. Hauser (2017) compared the morphological characters of more than 60 specimens of the two putative species from northern Thailand, and claimed *P. macularius* as a valid species, distinguishable from *P. margaritophorus* by the 7–13 rows of mid dorsal scales feebly keeled at midbody and the form and color of the nuchal collar. Our phylogenetic results showed that the species with color pattern I suggested by Zhao et al. (1998) were polyphyletic, with two distinct lineages including *P. margaritophorus* (dorsal scales smoothed) and *P. macularius* (dorsal scales keeled) (Figs 1–3). The average divergences of these two lineages were 15.5% (cyt b based) and 18.3% (ND4 based), indicating that separation occurred very early. Therefore, *P. macularius* should be considered a valid taxon.

It was noticed that both morphological comparisons and molecular analyses consistently showed that *Pareas* contained two distinct evolutionary lineages with distinguishable morphological differences and significant genetic divergences; however, the non-monophyly of *Pareas* was not well supported, and the loci used and specimens measured were limited. Whether *Pareas* should be split into two distinct genera needs more data to clarify.

Finally, a key to the species of *Pareas* is provide in Appendix 2.

## Supplementary Material

XML Treatment for Pareas menglaensis

XML Treatment for Pareas mengziensis

## Appendix 1

**Specimens morphologically examined in this study**

***Pareas formosensis***: Hainan, China (YBU 12015, YBU 12032, YBU 17029, R0263, R0542, R0543), Leishan, Guizhou, China (YBU 12090), Rongjiang, Guizhou, China (YBU 12115), Fangchenggang, Guangxi, China (YBU14508), Yanshan, Jiangxi, China (YBU 14573). ***P. mengziensis* sp. nov.**: Mengzi, Yunnan, China (YBU 14251, YBU 15252, YBU 14253, YBU 14288), Kaiyuan, Yunnan, China (YBU 15100, YBU 15114). ***P. boulengeri***: Chunan, Zhejiang, China (YBU 17155, YBU 17245), Wufeng, Hubei, China (YBU 13323A). ***P. chinensis***: Junlian, Sichuan, China (YBU 14126, YBU 16134, YBU 17043), Yingjing, Sichuan, China (YBU 16119, YBU 16122). ***P. stanleyi***: Leishan, Guizhou, China (YBU 12094). ***P. macularius***: Hainan, China (R0047, R0048, R0210, R0545, R0546, R0547, YBU 12016, YBU 17030), Jingdong, Yunnan, China (YBU 17062, YBU 17078). ***P. margaritophorus***: Cangwu, Guangxi, China (YBU 16061, YBU 16077, YBU 16095, YBU 17164). ***P. menglaensis* sp. nov.**: Mengla, Yunnan, China (YBU 14124, YBU 14141, YBU 14142).

## Appendix 2

**Key to *Pareas* species**

**Table:**

1	Two or three distinct narrow suboculars	**2**
–	One thin elongated subocular	**4**
2	Prefrontal bordering orbits, a large black blotch on the nape	***P. nuchalis***
–	Prefrontal separated from orbit, absence black blotch on the nape	**3**
3	3–5 rows of middle dorsal scales keeled	***P. carinatus***
–	9–13 rows of middle dorsal scales keeled	***P. menglaensis* sp. nov.**
4	Uniform purple brown or blue gray above with bicolored cross bars (color pattern I)	**5**
–	Light or dark brown above without bicolored dorsal scales (color pattern II)	**6**
5	All dorsal scales smooth	***P. macularius***
–	Dorsal scales keeled	***P. margaritophorus***
6	Loreal bordering orbit	**7**
–	Loreal separating from orbit	**10**
7	Vertebral scales enlarged	***P. monticola***
–	Vertebral scales not enlarged	**8**
8	Supralabials 6	***P. vindumi***
–	Supralabials 7 or 8	**9**
9	All dorsal scales smooth	***P. boulengeri***
–	Five rows of middle dorsal scales keeled	***P. stanleyi***
10	Dorsal scales not enlarged	***P. chinensis***
–	Dorsal scales enlarged	**11**
11	Three rows middle dorsal scales enlarged	**12**
–	Only vertebral scales enlarged	**14**
12	A large black area on the back of head and body	***P. mengziensis* sp. nov.**
–	Absence large black area on the back of head and body	**13**
13	Temporals 2+4, 5–9 rows middle dorsal scales keeled	***P. atayal***
–	Temporals 2+3 or 3+4, 9–13 rows middle dorsal scales keeled	***P. komaii***
14	Temporals 1+2	**15**
–	Temporals 2+3 or 3+4	**16**
15	The back of head purely black, postocular absent	***P. nigriceps***
–	The back of head pale brown with black spots, postocular 1	***P. hamptoni***
16	All dorsal scales smooth or 2 middle rows feebly keeled	***P. formosensis***
–	Middle dorsal scales keeled in rows 5–7	***P. iwasakii***

## Supplementary materials

Supplementary material 1 Appendix S1 Data type occurrence Explanation note Samples and sequences used in this study (BHNS: Bombay Natural History Society, Mumbai, India; CAS: California Academy of Science, San Francisco, USA; CES: Centre for Ecological Sciences, IISc, Bengaluru, India; FK: voucher listed by Kraus and Brown (1998); FMNH: Field Museum of Natural History, Chicago, USA; GP: P. Guo own catalogue number; HC: Cryobanking project, Taiwan, China; KIZ: Kunming Institute of Zoology, Chinese Academy of Sciences, Kunming, China; LSUHC: La Sierra University Herpetological Collection, Riverside, California, USA; NMNS: National Museum of Natural Science, Taiwan, China; YBU: Yibin University, Sichuan, China; YPX: Field number of KIZ). https://binary.pensoft.net/file/419710This dataset is made available under the Open Database License (http://opendatacommons.org/licenses/odbl/1.0/). The Open Database License (ODbL) is a license agreement intended to allow users to freely share, modify, and use this Dataset while maintaining this same freedom for others, provided that the original source and author(s) are credited.Ping Wang, Jing Che, Qin Liu, Ke Li, Jie Qiong Jin, Ke Jiang, Lei Shi, Peng Guo

Supplementary material 2 Appendix S2 Data type measurement Explanation note Characters recorded of *Pareas*. https://binary.pensoft.net/file/419711This dataset is made available under the Open Database License (http://opendatacommons.org/licenses/odbl/1.0/). The Open Database License (ODbL) is a license agreement intended to allow users to freely share, modify, and use this Dataset while maintaining this same freedom for others, provided that the original source and author(s) are credited.Ping Wang, Jing Che, Qin Liu, Ke Li, Jie Qiong Jin, Ke Jiang, Lei Shi, Peng Guo

Supplementary material 3 Appendix S3 Data type measurement Explanation note A comparison between holotype and paratypes of two new described species. https://binary.pensoft.net/file/419712This dataset is made available under the Open Database License (http://opendatacommons.org/licenses/odbl/1.0/). The Open Database License (ODbL) is a license agreement intended to allow users to freely share, modify, and use this Dataset while maintaining this same freedom for others, provided that the original source and author(s) are credited.Ping Wang, Jing Che, Qin Liu, Ke Li, Jie Qiong Jin, Ke Jiang, Lei Shi, Peng Guo

Supplementary material 4 Figure 1 Data type multimedia Explanation note The comparisons of anterior dorsal and head (column 1), lateral middle body (column 2) and subcaudal coloration (column 3) among Mengzi specimens and relatives. **A** Mengzi specimens **B***Pareas formosensis***C***P. chinensis***D***P. boulengeri***E***P. stanleyi*. https://binary.pensoft.net/file/419713This dataset is made available under the Open Database License (http://opendatacommons.org/licenses/odbl/1.0/). The Open Database License (ODbL) is a license agreement intended to allow users to freely share, modify, and use this Dataset while maintaining this same freedom for others, provided that the original source and author(s) are credited.Ping Wang, Jing Che, Qin Liu, Ke Li, Jie Qiong Jin, Ke Jiang, Lei Shi, Peng Guo
