# Supplementary material for: A revised taxonomy of Asian snail-eating snakes Pareas (Squamata, Pareidae): evidence from morphological comparison and molecular phylogeny
Source: Zookeys. 2020 Jun 9;939:45–64. doi: 10.3897/zookeys.939.49309 (PMC7297803; doi:10.3897/zookeys.939.49309)
Supplement: Supplementary material 1 — Appendix S1 [file zookeys-939-045-s001.docx]

**Appendix S1.** Samples and sequences used in this study (BHNS: Bombay Natural History Society, Mumbai, India; CAS: California Academy of Science, San Francisco, USA; CES: Centre for Ecological Sciences, IISc, Bengaluru, India; FK: voucher listed by Kraus and Brown (1998); FMNH: Field Museum of Natural History, Chicago, USA; GP: P. Guo own catalogue number; HC: Cryobanking project, Taiwan, China; KIZ: Kunming Institute of Zoology, Chinese Academy of Sciences, Kunming, China; LSUHC: La Sierra University Herpetological Collection, Riverside, California, USA; NMNS: National Museum of Natural Science, Taiwan, China; YBU: Yibin University, Sichuan, China; YPX: Field number of KIZ).

| Taxon | Voucher number | Locality | GenBank accession number | | | | |
| --- | --- | --- | --- | --- | --- | --- | --- |
|  |  |  | cyt b | c-mos | ND4 | Rag1 | Source |
| *Pareas formosensis* | YBU 12015 | Hainan, China | MK135068 | MK135117 | MK805333 | MK805385 | This study |
| *P. formosensis* | GP 2164 | Hainan, China | MK135069 | MK135118 | MK805334 | MK805386 | This study |
| *P. formosensis* | GP 2165 | Hainan, China | MK135070 | MK135119 | MK805335 | MK805387 | This study |
| *P. formosensis* | YBU 12032 | Hainan, China | MK135071 | MK135120 | MK805336 | MK805388 | This study |
| *P. formosensis* | GP 4581 | Jingning, Zhejiang, China | MK135072 | MK135121 | MK805337 | MK805389 | This study |
| *P. formosensis* | YBU 17029 | Hainan, China | MK135073 | MK135122 | MK805338 | MK805390 | This study |
| *P. formosensis* | YBU 12090 | Leishan, Guizhou, China | MK135074 | MK135123 | MK805339 | MK805391 | This study |
| *P. formosensis* | YBU 12115 | Rongjiang, Guizhou, China | MK135075 | MK135124 | MK805340 | MK805392 | This study |
| *P. formosensis* | YBU 14508 | Guangxi, China | MK135076 | MK135125 | MK805341 | MK805393 | This study |
| *P. formosensis* | GP 3696 | Yanshan, Jiangxi, China | HM46857 | MK135166 | MK805382 | MK805434 | This study |
| *P. formosensis* | GP 3808 | Yanshan, Jiangxi, China | HM46858 | MK135167 | MK805383 | MK805435 | This study |
| *P. formosensis* | YBU 14573 | Yanshan, Jiangxi, China | HM46859 | MK135168 | MK805384 | MK805436 | This study |
| *P. formosensis 1* | NMNS 05632 | N. Cross-Is. Highway, Taiwan, China | KJ642130 | KJ642201 | – | – | You et al. 2015 |
| *P. formosensis 2* | NMNS 05637 | Xitou, Nantou, Taiwan, China | KJ642136 | KJ642202 | – | – | You et al. 2015 |
| *P. hamptoni* | YPX 18219 | Myanmar | MK135077 | MK135126 | MK805342 | MK805394 | This study |
| *P. hamptoni* | YPX 18604 | Myanmar | MK135078 | MK135127 | MK805343 | MK805395 | This study |
| *P. mengziensis* **sp. nov.** | GP 1294 | Mengzi, Yunnan, China | MK135079 | MK135128 | MK805344 | MK805396 | This study |
| *P. mengziensis* **sp. nov.** | YBU 14251 | Mengzi, Yunnan, China | MK135080 | MK135129 | MK805345 | MK805397 | This study |
| *P. mengziensis* **sp. nov.** | YBU 14252 | Mengzi, Yunnan, China | MK135081 | MK135130 | MK805346 | MK805398 | This study |
| *P. mengziensis* **sp. nov.** | YBU 14253 | Mengzi, Yunnan, China | MK135082 | MK135131 | MK805347 | MK805399 | This study |

**Appendix S1.** (continued)

| Taxon | Voucher number | Locality | GenBank accession number | | | | |
| --- | --- | --- | --- | --- | --- | --- | --- |
|  |  |  | cyt b | c-mos | ND4 | Rag1 | Source |
| *P. mengziensis* **sp. nov.** | YBU 14288 | Mengzi, Yunnan, China | MK135083 | MK135132 | MK805348 | MK805400 | This study |
| *P. mengziensis* **sp. nov.** | YBU 15100 | Kaiyuan, Yunnan, China | MK135084 | MK135133 | MK805349 | MK805401 | This study |
| *P. mengziensis* **sp. nov.** | YBU 15114 | Kaiyuan, Yunnan, China | MK135085 | MK135134 | MK805350 | MK805402 | This study |
| *P. stanleyi* | GP 229 | Guangxi, China | MK135086 | MK135135 | MK805351 | MK805403 | This study |
| *P. stanleyi* | YBU 12094 | Leishan, Guizhou, China | MK135087 | MK135136 | MK805352 | MK805404 | This study |
| *P. chinensis* | GP 2196 | Junlian, Sichuan, China | MK135088 | MK135137 | MK805353 | MK805405 | This study |
| *P. chinensis* | GP 2383 | Hongya, Sichuan, China | MK135089 | MK135138 | MK805354 | MK805406 | This study |
| *P. boulengeri* | GP 2923 | Jiangkou, Guizhou, China | MK135090 | MK135139 | MK805355 | MK805407 | This study |
| *P. boulengeri* | GP 207 | Anxian, Sichuan, China | MK135091 | MK135140 | MK805356 | MK805408 | This study |
| *P. boulengeri* | YBU 13323A | Wufeng, Hubei, China | MK135092 | MK135141 | MK805357 | MK805409 | This study |
| *P. boulengeri* | GP 4716 | Yidu, Hubei, China | MK135093 | MK135142 | MK805358 | MK805410 | This study |
| *P. boulengeri* | GP 3428 | Yixian, Anhui, China | MK135094 | MK135143 | MK805359 | MK805411 | This study |
| *P. boulengeri* | YBU 17155 | Chunan, Zhejiang, China | MK135095 | MK135144 | MK805360 | MK805412 | This study |
| *P. boulengeri* | YBU 17245 | Chunan, Zhejiang, China | MK135096 | MK135145 | MK805361 | MK805413 | This study |
| *P. margaritophorus* | YBU 16061 | Cangwu, Guangxi, China | MK135097 | MK135146 | MK805362 | MK805414 | This study |
| *P. margaritophorus* | YBU 17164 | Cangwu, Guangxi, China | MK135098 | MK135147 | MK805363 | MK805415 | This study |
| *P. margaritophorus* | GP 4437 | Cangwu, Guangxi, China | MK135099 | MK135148 | MK805364 | MK805416 | This study |
| *P. margaritophorus* | YBU 16095 | Cangwu, Guangxi, China | MK135100 | MK135149 | MK805365 | MK805417 | This study |
| *P. macularius* | GP815 | Hainan, China | MK135101 | MK135150 | MK805366 | MK805418 | This study |
| *P. macularius* | GP 2110 | Hainan, China | MK135102 | MK135151 | MK805367 | MK805419 | This study |
| *P. macularius* | YBU 12016 | Hainan, China | MK135103 | MK135152 | MK805368 | MK805420 | This study |
| *P. macularius* | YBU 17030 | Hainan, China | MK135104 | MK135153 | MK805369 | MK805421 | This study |
| *P. macularius* | YBU 17078 | Jingdong, Yunnan, China | MK135105 | MK135154 | MK805370 | MK805422 | This study |
| *P. macularius* | YBU 17062 | Jingdong, Yunnan, China | MK135106 | MK135155 | MK805371 | MK805423 | This study |

**Appendix S1.** (continued)

| Taxon | Voucher number | Locality | GenBank accession number | | | | |
| --- | --- | --- | --- | --- | --- | --- | --- |
|  |  |  | cyt b | c-mos | ND4 | Rag1 | Source |
| *P. monticola* | GP 2027 | Motuo, Xizang, China | MK135107 | MK135156 | MK805372 | MK805424 | This study |
| *P. monticola* | KIZ 047036 | Pingbian, Yunnan, China | MK135108 | MK135157 | MK805373 | MK805425 | This study |
| *P. monticola* | KIZ 014167 | Motuo, Xizang, China | MK135109 | MK135158 | MK805374 | MK805426 | This study |
| *P. carinatus* | GP 1079 | Malaysia | MK135110 | MK135159 | MK805375 | MK805427 | This study |
| *P. carinatus* | KIZ 011972 | Malaysia | MK135111 | MK135160 | MK805376 | MK805428 | This study |
| *P. carinatus* | KIZ 011970 | Malaysia | MK135112 | MK135161 | MK805377 | MK805429 | This study |
| *P. menglaensis* **sp. nov.** | GP 1292 | Mengla, Yunnan, China | MK135113 | MK135162 | MK805378 | MK805430 | This study |
| *P. menglaensis* **sp. nov.** | YBU 14124 | Mengla, Yunnan, China | MK135114 | MK135163 | MK805379 | MK805431 | This study |
| *P. menglaensis* **sp. nov.** | YBU 14141 | Mengla, Yunnan, China | MK135115 | MK135164 | MK805380 | MK805432 | This study |
| *P. menglaensis* **sp. nov.** | YBU 14142 | Mengla, Yunnan, China | MK135116 | MK135165 | MK805381 | MK805433 | This study |
| *P. atayal1* | HC 000618 | N. Cross-Is. Highway, Taiwan, China | JF827685 | JF827711 | JF827662 | – | Guo et al. 2011 |
| *P. atayal2* | HC 000628 | N. Cross-Is. Highway, Taiwan, China | JF827686 | JF827712 | JF827663 | – | Guo et al. 2011 |
| *P. komaii1* | HC 000669 | Lijia, Taidong, Taiwan, China | JF827687 | JF827713 | JF827664 | – | Guo et al. 2011 |
| *P. komaii2* | NMNS 05598 | Daxueshan, Taichung, Taiwan, China | KJ642162 | KJ642210 | – | – | You et al. 2015 |
| *P. iwasakii1* | I03-ISG1 | Ishigaki Is., S. Ryukyu, Japan | KJ642158 | KJ642207 | – | – | You et al. 2015 |
| *P. iwasakii2* | I04-ISG2 | Ishigaki Is., S. Ryukyu, Japan | KJ642159 | KJ642208 | – | – | You et al. 2015 |
| *P. macularius* | CAS 206620 | Bago Division, Myanmar | AF471082 | AY471150 | – | – | Guo et al. 2011 |
| *P. nuchalis* | FK 2626 | Belait, Brunei | – | – | U49311 | – | Kraus and Brown, 1998 |
| *Aplopeltura boa1* | KIZ 011963 | Malaysia | JF827673 | JF827696 | JF827650 | – | Guo et al. 2011 |
| *A. boa2* | LSUHC 7248 | Malaysia | KC916746 | AF544715 | U49312 | – | Loredo et al. 2013/Vidal and Hedges, 2002/Kraus and Brown, 1998 |
| *Asthenodipsas laevis* | LSUHC 10346 | Peninsular Malaysia | KC916749 | KX660335 | KX660596 | – | Loredo et al. 2013/Figueroa et al. 2016 |

**Appendix S1.** (continued)

| Taxon | Voucher number | Locality | GenBank accession number | | | | |
| --- | --- | --- | --- | --- | --- | --- | --- |
|  |  |  | cyt b | c-mos | ND4 | Rag1 | Source |
| *A. malaccanus* | FMNH 273617 | – | KX660469 | KX660336 | KX660597 | – | Figueroa et al. 2016 |
| *A. vertebralis1* | LSUHC 9138 | Peninsular Malaysia | KC916754 | – | – | – | Loredo et al. 2013 |
| *A. vertebralis2* | LSUHC 9873 | Peninsular Malaysia | KC916753 | – | – | – | Loredo et al. 2013 |
| *A. lasgalenensis1* | 10668 | Peninsular Malaysia | KC916776 | – | – | – | Loredo et al. 2013 |
| *A. lasgalenensis2* | 7228 | Peninsular Malaysia | KC916769 | – | – | – | Loredo et al. 2013 |
| *A. tropidonotus* |  | Sumatra, Indonesia | AY425808 | – | – | – | Fernandes and Malhotra (unpublished) |
| *Xylophis. perroteti* | CES 2016b | Nilgiris, Tamil Nadu, India | – | MK344193 | MK340910 | MK340913 | Deepak et al. 2018 |
| *X. stenorhynchus* | CAS 17199 | India | MK340915 | MK344194 | MK340911 | – | Deepak et al. 2018 |
| *X. captaini* | BNHS 3376 | Kottayam, Kerala, India | MK340914 | MK344195 | MK340912 | – | Deepak et al. 2018 |
| *Lycodon rufozonatum* | GP 625 | Dandong, Liaoning, China | KC733196 | KC733213 | KC733229 | KC733181 | Guo et al. 2013 |
| *Gloydius brevicaudus* | GP1099 | Dalian, Liaoning, China | JQ687497 | JQ687516 | JQ687478 | – | Guo et al. 2012 |
| *Xenodermus javanicus* | FMNH 230073 | Malaysia | AY425810 | AF544711 | U49320 | – | Fernandes and Malhotra (unpublished)/Vidal and Hedges, 2002/Kraus and Brown, 1998 |
