# Supplementary material for: A revised taxonomy of Asian snail-eating snakes Pareas (Squamata, Pareidae): evidence from morphological comparison and molecular phylogeny
Source: Zookeys. 2020 Jun 9;939:45–64. doi: 10.3897/zookeys.939.49309 (PMC7297803; doi:10.3897/zookeys.939.49309)
Supplement: Supplementary material 2 — Appendix S2 [file zookeys-939-045-s002.docx]

**Appendix S2.** Characters recorded of *Pareas*.

| Taxa | LCs | SubO | PrFBO | LoBO | SPOF | PreO | PosO | Tem | NED | NKD |
| --- | --- | --- | --- | --- | --- | --- | --- | --- | --- | --- |
| *Pareas menglaensis* **sp. nov.** | 3 | 2 | - | - | - | 2 | 1 | 3+4/3+3 | 3 | 5/11/11 |
| *P. carinatus* | 3 | 2 or 3 | - | - | - | 1 or 2 | 1 or 2 | 2+3/3+4 | 3 | -/3/- |
| *P. nuchalis* | 3 | 3 | + | - | - | 2 | 2 | 3+3/3+4 | 1 | -/9~13/- |
| *P. margaritophorus* | 1 | 1 | + | - | -/+ | 1 | 1 or 0 | 2+3/2+3 | 0 | 0 |
| *P. macularius* | 1 | 1 | + | - | -/+ | 1 | 1 or 0 | 2+3/2+2 | 0 | 3~5/3~9/7~11 |
| *P. hamptoni* | 1 | 1 | + | - | + | 2 | 0 | 1+2 | - | feebly keeled |
| *P. mengziensis* **sp. nov.** | 1 | 1 | + | - | + | 1 | 0 | 2+3 | 3 | 3~5/5~7/7~9 |
| *P. formosensis* | 1 | 1 | + | - | +/- | 1 | 1 or 0 | 2+3, few 2+2 | 1 | 0/0~2/0~4 |
| *P. atayal* | 1 | 1 | + | - | - | 1 | 1 | 2+4 | 3 | 0~3/7~9/7~9 |
| *P. iwasakii* | 1 | 1 | + | - | - | 1 | 1 | 3+4 or 2+3 | 1 | 0~3/3~7/7~9 |
| *P. komaii* | 1 | 1 | + | - | - | 1 | 1 | 3+4 or 2+3 | 3 | 0~9/9~13/9~13 |
| *P. chinensis* | 1 | 1 | + | - | + | 1 | 0 | 2+3, rarely 3+3 | 0 | 5/5~7/7~9 |
| *P. nigriceps* | 1 | 1 | + | - | + | 1 | 0 | 1+2 | 1 | 0/9/9 |
| *P. boulengeri* | 1 | 1 | + | + | + | 0 | 0 | 2+3 | 0 | 0 |
| *P. stanleyi* | 1 | 1 | + | + | + | 0 | 0 | 2+3 | 0 | 0/5/5-13 |
| *P. vindumi* | 1 | 1 | + | + | + | 1 | 0 | 2+3 | 0 | 0 |
| *P. monticola* | 1 | 1 | + | + | - | 0 | 1 | 1+2 or 2+3 | 1 | feebly keeled |

Abbreviations: InfL: infralabials; LCs: the largest chin-shield pairs; LoBO: loreal bordering orbit; Max: maxillary; NED: number of enlarged dorsal scale rows at midbody; NKD: number of keeled dorsal scale rows at anterior/middle/posterior of body; PosO: postoculars; PreO: preoculars; PrFBO: prefrontal bordering orbit; Sc: subcaudals; SPOF: subocular-postocular fused or not; SubO: suboculars; SupL: supralabials; Tem: temporals; Vs: ventrals.

**Appendix S2.** (continued)

| Taxa | Vs | Sc | TL/SVL | Max | SupL | InfL |
| --- | --- | --- | --- | --- | --- | --- |
| *Pareas menglaensis* **sp. nov.** | ♀177, ♂176 | ♀65, ♂74~79 | ♀0.24, ♂0.28~0.31 | 4~5 | 7 | 7~8 |
| *P. carinatus* | ♀162, ♂163~178 | ♀57, ♂65~80 | ♀0.23 | - | 7~8 | - |
| *P. nuchalis* | 195~213 | 105~113 | ♂0.26 | - | 8~9 | - |
| *P. margaritophorus* | 138 | 53 | ♂0.29 | 5 | 6~7 | 6 or 7 |
| *P. macularius* | ♀152~162, ♂149~164 | ♀40~41, ♂46~53 | ♀0.17, ♂0.21~0.27 | 4~6 | 6~7 | 6~9 |
| *P. hamptoni* | ♂202 | ♂96 | ♂0.27 | - | 7~8 | - |
| *P.* mengziensis **sp. nov.** | ♀169~173, ♂167~169 | ♀54~60, ♂54~61 | ♀0.22~0.23, ♂0.25~0.26 | 6~7 | 7, rarely 6 | 8~9 |
| *P. formosensis* | ♀183~195, ♂181~193 | ♀82~89, ♂81~93 | ♀0.25~0.30, ♂0.29~0.33 | 6~7, rarely 5 or 8 | 6~8 | 7~8, few 9 |
| *P. atayal* | 174~188 | 71~77 | ♀0.22 | 6~7 | 7 | 7~9 |
| *P. iwasakii* | 189~194 | 76~84 | 0.21-0.24 | - | - | 9~11 |
| *P. komaii* | 162~182 | 60~76 | 0.20-0.25 | - | - | 6~9 |
| *P. chinensis* | ♀176, ♂172~176 | ♀69, ♂76~82 | ♀0.27, ♂0.34~0.36 | 5~6 | 7~8 | 8~9 |
| *P. nigriceps* | ♀175 | ♀76 | ♀0.32 | 6~7 | 7 | 7 |
| *P. boulengeri* | ♀186, ♂178~183 | ♀69, ♂69~75 | ♀0.22, ♂0.27~0.28 | 5~6 | 7 | 7~8 |
| *P. stanleyi* | 152~186 | 45~77 | ♀0.17, ♂0.20 | 5 | 7 | 8 |
| *P. vindumi* | ♀178 | ♀61 | ♀0.25 | - | 6 | 6 |
| *P. monticola* | ♀181~188, ♂184~196 | ♀72, ♂77~86 | ♀0.23, ♂0.18 | 4~9 | 7~8 | 7~8 |
