# Supplementary material for: A revised taxonomy of Asian snail-eating snakes Pareas (Squamata, Pareidae): evidence from morphological comparison and molecular phylogeny
Source: Zookeys. 2020 Jun 9;939:45–64. doi: 10.3897/zookeys.939.49309 (PMC7297803; doi:10.3897/zookeys.939.49309)
Supplement: Supplementary material 3 — Appendix S3 [file zookeys-939-045-s003.docx]

**Appendix S3. A comparison between holotype and paratypes of two new described species.**

|  | *Pareas menglaensis* **sp. nov.** | | | *Northpareas mengziensis* **sp. nov.** | | | | | |
| --- | --- | --- | --- | --- | --- | --- | --- | --- | --- |
|  | Holotype | Paratypes | | Holotype | Paratypes | | | | |
|  | YBU 14124 | YBU 14141 | YBU 14142 | YBU14252 | YBU14251 | YBU15100 | YBU14253 | YBU14288 | YBU15114 |
| SEX | ♀ | ♂ | ♂ | ♀ | ♀ | ♀ | ♂ | ♂ | ♂ |
| SVL | 472 | 448 | 353 | 426 | 422 | 201 | 355 | 473 | 340 |
| TL | 111 | 137 | 98 | 98 | 91 | 46 | 94 | 94 | 85 |
| PrFBO | No | No | No | Yes | Yes | Yes | Yes | Yes | Yes |
| PreO | 2 | 2 | 2 | 1 | 1 | 1 | 1 | 1 | 1 |
| PosO | 1 | 1 | 1 | 0 | 0 | 0 | 0 | 0 | 0 |
| SubO | 2 | 2 | 2 | 1 | 1 | 1 | 1 | 1 | 1 |
| Tem | 3+4+3/3+3+3 | 3+4+3 | 3+4+4 | 2+3+3 | 2+3+3 | 2+3+3 | 2+3+3 | 2+3+3 | 2+3+3 |
| SupL | 7/7 | 7/7 | 7/7 | 7 | 7 | 7 | 7 | 7 | 7 |
| InfL | 7(4)/8(4) | 8/7 | 7/8 | 9 | 8 | 9/8 | 8/9 | 8 | 8 |
| LCs | 3 | 3 | 3 | 1 | 1 | 1 | 1 | 1 | 1 |
| LoBO | Yes | Yes | Yes | No | No | No | No | No | No |
| Vs | 177 | 176 | 176 | 170 | 169 | 173 | 169 | 167 | 168 |
| Sc | 65 | 74 | 74 | 54 | 55 | 59 | 61 | 54 | 55 |
| Ds | 15-15-15 | 15-15-15 | 15-15-15 | 15-15-15 | 15-15-15 | 15-15-15 | 15-15-15 | 15-15-15 | 15-15-15 |
| NED | 3 | 3 | 3 | 3 | 3 | 3 | 3 | 3 | 3 |
| NKD | 11 | 11 | 11 | 5 | 5 | 3 | 7 | 7 | 7 |
| Max | 5 | 3/4 | 4/3 | 6/6 | 6/6 | 6/6 | 7/7 | 7/7 | 7/7 |

**Abbreviations:** SVL: snot-vent length; TL: tail length; PrFBO: prefrontal bordering orbit; PreO: preoculars; PosO: postoculars; SubO: suboculars; Tem: temporals; SupL: supralabials; InfL: infralabials; LCs: the largest chin-shield pairs; LoBO: loreal bordering orbit; Vs: ventral scales; Sc: subcaudals; Ds: dorsal scales; NED: number of enlarged dorsal scale rows at midbody; NKD: number of keeled dorsal scale rows at anterior/middle/posterior of body; Max: maxillary.
