# Supplementary figures and images for: A revised taxonomy of Asian snail-eating snakes Pareas (Squamata, Pareidae): evidence from morphological comparison and molecular phylogeny
Source: Zookeys. 2020 Jun 9;939:45–64. doi: 10.3897/zookeys.939.49309 (PMC7297803; doi:10.3897/zookeys.939.49309)

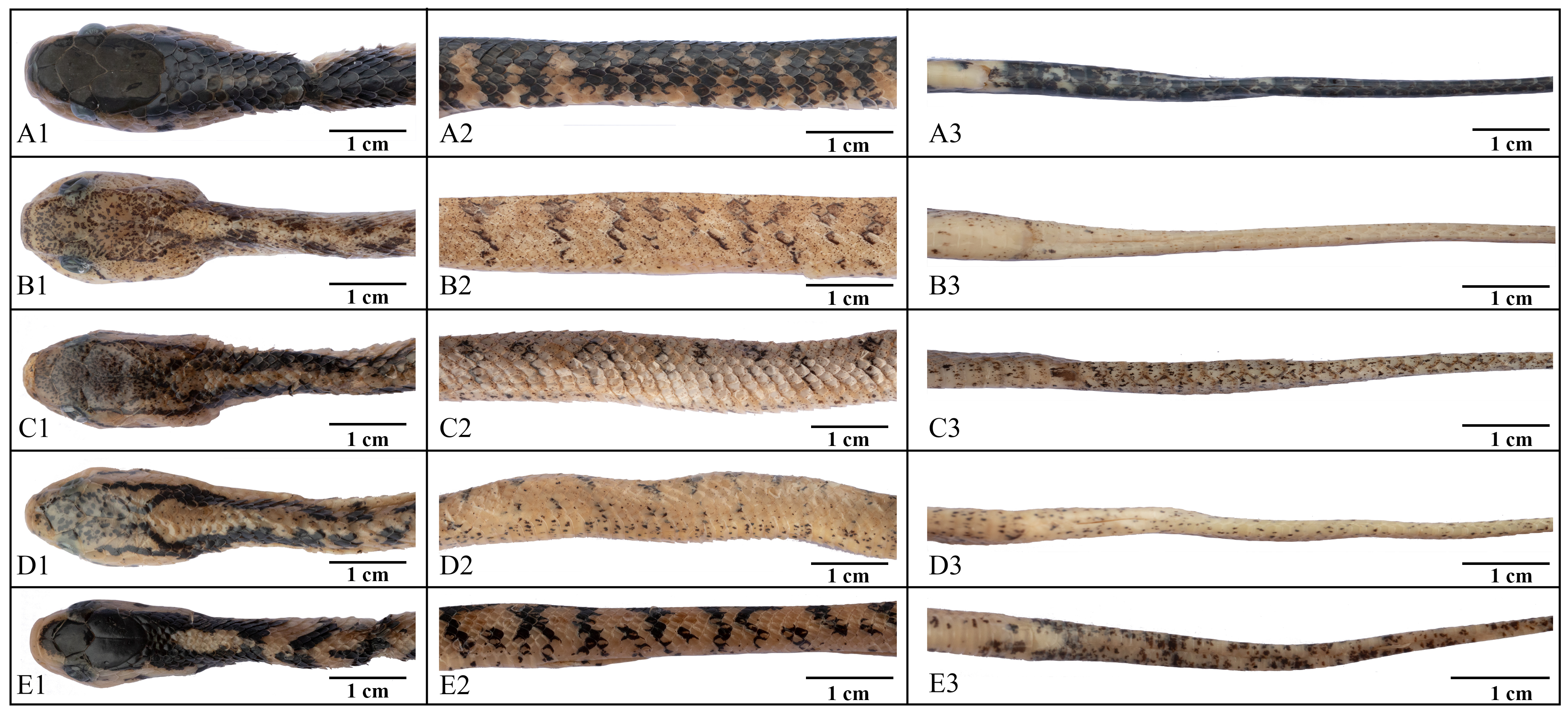

Supplement: Supplementary material 4 — Figure 1 [file zookeys-939-045-s004.jpg]
